# Investigation of Alternative Therapeutic and Biocidal Options to Combat Antifungal-Resistant Zoonotic Fungal Pathogens Isolated from Companion Animals

**DOI:** 10.3390/idr13020034

**Published:** 2021-04-11

**Authors:** Elaine Meade, Micheal Savage, Mark Slattery, Mary Garvey

**Affiliations:** 1 Department of Life Science, Institute of Technology, Ash Lane, F91YW50 Sligo, Ireland; Elaine.Meade@mail.itsligo.ie; 2 Lir Analytical LTD, Century Business Park, Unit 2, Athlone Rd, N39Y935 Longford, Ireland; micheal.savage@liranalytical.com; 3 Mark Anthony Slattery, Veterinary Practice, F91DP62 Manorhamilton, Ireland; markslats@gmail.com

**Keywords:** fungal skin infections, pathogenicity, resistance, zoonotic, spore formers

## Abstract

Fungal skin infections and iatrogenic disease of companion animals continue to be an ongoing issue for veterinarians, where misdiagnosis or inapt medical treatment result in secondary conditions within animals. The widespread use of antifungals in both modern medicine and agriculture has resulted in concomitant resistance in species, where zoonotic transfer poses a risk to public health. Studies described herein assess the resistance of pathogenic species isolated from companion animals to a battery of conventional antimicrobial agents. Levels of resistance were detected using recognised in vitro methods, where additional novel therapeutic and biocide options were also extensively investigated. Results show high levels of resistance to the three main families of antifungal agents, namely caspofungin, Amp B and fluconazole. Resistance in *Candida*, *Cryptococcal*, *Aspergillus* and *Trichophyton* species is described herein, highlighting the need for defined species-specific antifungal breakpoints, and for *Malassezia* and *Wickerhamomyces anomalus* species which also have zoonotic potential. Novel compound phendione showed promising antimicrobial activity, with MICs determined for both fungal and bacterial species. The biocidal options investigated also showed potential to act as intermediate-level disinfectants, where peracetic acid proved most effective against fungal spore formers.

## 1. Introduction

Animal mycoses is a common reoccurring issue globally, with zoonosis a risk in companion animals, where humans are continually exposed to infectious propagules [1]. Dermal infections are typically caused by dermatophytes requiring keratin for growth such as *Microsporum*, *Trichophyton*, and *Epidermophyton* species. Superficial zoophilic skin infections (dermatomycosis) are also caused by non-dermatophyte fungal species such as *Malassezia*, *Aspergillus* and *Candida* (benign colonisers of mucosal surfaces). Dermal tissue may become infected following colonisation of the cutaneous tissue and dermal follicles with opportunistic and pathogenic fungi. 

Invasive and systemic fungal disease can result from movement of the fungal species transdermally via open wounds [2]. Systemic fungal diseases cause significant and often severe morbidity and fatality in companion animals. The mortality rate following infection with invasive fungal infections remains high, particularly when caused by rare yeast pathogens [3]. Immunosuppression and iatrogenic disease are issues in both human and animal patients, with increasing prevalence globally. This has also promoted the emergence of opportunistic fungal infections which previously occurred only rarely [4] and is a serious public health risk to the immunocompromised population. 

### 1.1. Mycosis Species

*Wickerhamomyces anomalus* (formerly *Pichia anomala*) is a zoonotic and nosocomial yeast, associated with the production of lethal mycotoxins having a mortality rate of 38% in adults and 42% in paediatric patients globally [5]. Several case reports describe disseminated candidiasis in dogs, with *Candida parapsilosis* associated with fungal pneumonia, urinary tract, and dermal infections [6] and is one of the most common fungus associated with respiratory disease in humans [7]. Zoonotic *Cryptococcus* is also predominately associated with lung infection in immunocompromised patients, with dissemination to the brain causing meningoencephalitis, afflicting approximately 1 million AIDS patients, with 600,000 deaths annually [8]. Importantly, zoonotic, and zoophilic fungal infections tend to be more inflammatory than those caused by anthropophilic fungi transmitted from person to person [9]. Accurately diagnosing animal fungal infections is highly important to determine the prognosis, and therapeutic options, and to prevent zoonotic transmission, particularly as species differ greatly in their susceptibility to antifungal drugs. *Aspergillus* infections, for example, cannot be differentiated clinically from infections caused by other pathogens requiring a different therapeutic approach and posing a different zoonotic risk [10]. 

### 1.2. Human Mycosis

Fungal infections in humans are treated with intravenous amphotericin B (AMP B), oral azoles, and oral flucytosine. The safety and efficacy of these antifungals in animals is unknown. However, AMP B does induce nephrotoxicity in canines [11]. Furthermore, antifungal resistance is commonplace in fungal species both intrinsically and acquired—for example, flucytosine has limited antifungal activity. Fluconazole, which is more hydrophilic, smaller in size and less prone to protein binding is a possible treatment option. To date, treatment of canine systemic mycosis is largely unsuccessful, with newer triazole agents having some success [12] but limited application due to cost issues as treatment is prolonged and often lifelong [10]. Additionally, antifungal susceptibility testing is predominantly only conducted on strains causing invasive infections, and is largely restricted to *Malassezia furfur* and *Malassezia pachydermatis*. There are no standardised clinical breakpoints in place for these *Malassezia* species. There is a need for additional therapeutic and biocidal options to combat disseminated canine fungal infections and to prevent environmental and zoonotic transmission. This study aims to investigate the antifungal resistance of various fungal species isolated from chronic cases of infection in canine companion animals. Phendione (1,10-phenanthroline-5,6-dione), a phenanthrene-based ligand [13], which has demonstrated antimicrobial efficacy against *Pseudomonas* species [14] and *Candida* isolates [15], will be assessed for antifungal activity against all isolated species in this study. Studies will also determine suitable biocidal options for controlling environmental contamination and reducing zoonotic transmission of each species.

## 2. Results

Table 1 displays the antibiogram for the seven bacterial co-isolates of canine dermal cases of infection (see case descriptions for patients 3, 4, 5 and 7) where all bacterial species showed adequate levels of sensitivity to the various drug classes. This is of importance as antibiotic therapy is administered for such cases of infection without diagnostic analysis. Amox/clav is the most commonly prescribed empirical treatment option in both veterinary and human medicine, with all test species proving susceptible to the penicillin class, with the exception of *Enterobacter asburiae* isolate. Further testing demonstrated that this species was sensitive to the third-generation cephalosporins, where cefpodoxime provided a zone of 26 mm and ceftriaxone and cefotaxime provided a respective zone of 27 mm. The cephalosporin class additionally demonstrated activity against *Staphylococcal* species, *Streptococcus canis* and *Proteus mirabilis*, with significant zone diameters ranging from 20 to 35 mm, where the *Enterococcus* species naturally exert a low-level intrinsic resistance to βeta lactams. This is due to the fact that enterococci possess penicillin-binding proteins (PBPs) with a low affinity for these antibiotics. Albeit, these agents can be used in combination with other drug classes to treat severe infections, such as meningitis or endocarditis, where their synergistic effects with the aminoglycosides have shown to exert bactericidal activity. All Gram-positive test species proved sensitive to the critically important vancomycin, including the *Enterococcus* isolates, where vancomycin is one of the few available therapies for serious enterococcal infections. Notably, glycopeptides have little use against Gram-negative species, being unable to penetrate their outer membrane barrier. On the other hand, the *Enterococcus* species showed varying susceptibility to the carbapenem class, with both strains displaying resistance to doripenem and the *E. hirae* species further showing resistance to meropenem while remaining sensitive to imipenem, with a zone diameter of 22 mm observed, whereas the *E. faecium* isolate was resistant to imipenem but susceptible to meropenem producing a zone of 20 mm. All other test strains produced significant zones of inhibition for the carbapenems, where antibiotics in this category are reserved for last resort in human medicine and not authorised for use in the EU as veterinary medicine; being banned for use in food-producing animals and only administered to company animals under certain exceptional circumstances. The DNA-inhibiting quinolones and protein-inhibiting chloramphenicol drug classes proved to be universally effective against all test species, producing significant zones of inhibition ranging from 14 to 35 mm. The tetracycline and aminoglycoside classes were also successful options for all species, with few exceptions, where *P. mirabilis* was resistant to doxycycline and *E. hirae* was resistant to streptomycin, with *E. faecium* displaying reduced sensitivity to the aminoglycoside with a zone of 12 mm. The macrolide group was unsuccessful and ineffective against both Gram-negative species and the Gram-positive *Staphylococcus hominis* species, where outright resistance is observed for both azithromycin and erythromycin, with all other test species being susceptible to the bacteriostatic drugs. The monobactam and polymyxin class are listed by the WHO as critically important antimicrobials, being crucial last resort options for treatment of multidrug-resistant (MDR) Gram-negative infections. Antibiotic agents in these groups are inactive against Gram-positive aerobes and anaerobes, where aztreonam possesses a strong affinity for PBP3 present in Gram-negative bacteria and colistin binds LPS, disrupting the integrity of the outer membrane. Aztreonam demonstrated activity against both Gram-negative strains, with significant zone diameters of 30 mm produced, respectively, for *P. mirabilis* and *E. asburiae*. Colistin also proved effective against *E. asburiae*, producing a zone of 13 mm at 10 µg, which increased to 16 mm as the concentration increased to 50 µg, where *Proteus* species show intrinsic resistance to this drug type. 

**Figure 1:**
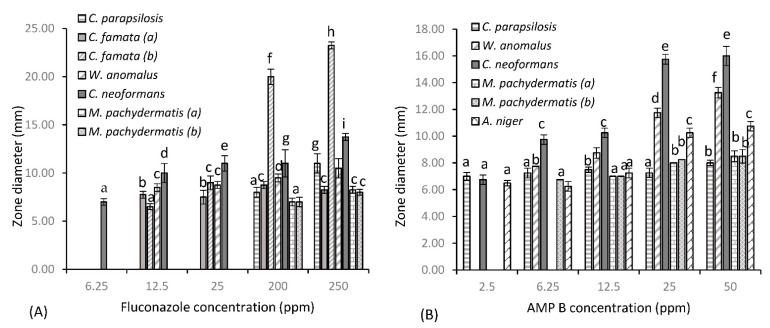

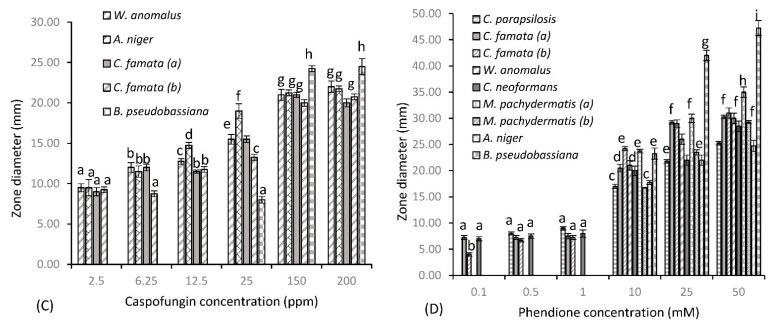
Zones of diameter (mm) for fungal dermal isolates of canine disease for (**A**) fluconazole, (**B**) AMP B, (**C**) caspofungin and (**D**) test agent phendione (±S.D). Alphabetical letters denote significant difference at *p* < 0.05.

Figure 1 shows the susceptibility of fungal test species to three common antifungal drugs and test agent phendione, as determined by the Kirby–Bauer assay. Fluconazole (Figure 1a) provided varying levels of inhibition among test species, with minimal activity seen at the lower concentrations of 6.25, 12.5 and 25 µg/mL. However, greater levels of inhibition were achieved at the higher concentrations of 200 and 250 µg/mL, where significant zones of 20 and 23.25 mm are observed, respectively, for *C. famata* (b). *C. neoformans* was the next most sensitive test organism, with a consistent zone of 11 mm produced at 25 and 200 µg/mL, which increased slightly to 13.75 mm at 250 µg/mL. Both *C. parapsilosis* and *W. anomalus* showed reduced sensitivity to fluconazole at the lower concentrations, where maximal zones of 11 and 10.5 mm were provided, respectively, at 250 µg/mL. Fluconazole’s spectrum of activity decreased significantly for all other test species, with little activity provided against *C. famata* (a) and both *M. pachydermatis* isolates, with the higher concentration providing minute zones of 8.25 mm. No zone was produced for these species up to concentrations of 25 µg/mL. This was true also for *Aspergillus niger* and *Beauveria pseudobassiana* (data not shown), where further increases in concentration failed to provide any zone of inhibition. *B. pseudobassiana* further proved resistant to AMP B, along with both *C. famata* isolates (data not shown), with no zone observed at all concentrations tested. *C. parapsilosis* displayed some resistance to AMP B, where inhibition plateaued across the concentration gradient, only varying by 1 mm (from 7 to 8 mm), showing that higher concentrations do not provide increased cell death for this strain. AMP B also proved less than effective against both lipophilic *Malassezia* species, where mirroring zones of 7, 8 and 8.50 mm were observed at 12.5, 25 and 50 µg/mL, respectively. On the other hand, *A. niger*, *W. anomalus* and *C. neoformans* showed greater sensitivity to AMP B treatment, and in that order (from lowest to highest). For these test species, dose-dependent increases in concentration resulted in an increase in zone diameter, with significant zones of 10.75, 13.25 and 16 mm, respectively, achieved for *A. niger*, *W. anomalus* and *C. neoformans* at a dose of 50 µg/mL. Caspofungin (Figure 1c) provided the greatest level of inhibition of all antifungal agents for susceptible test species. *W. anomalus*, *A. niger* and *C. famata* species all showed similar patterns of inhibition at the lower concentration of 2.5 µg/mL, with average zones of 9 mm observed. An increase in concentration to 6.25 µg/mL resulted in a zone increase for all these isolates to 12 mm, except for *C. famata* (b), where a concentration of 12.5 µg/mL was required to obtain similar results. *W. anomalus* and *C. famata* (b) remained relatively constant at 12.5 µg/mL, with a concentration of 25 µg/mL increasing zone diameters to 15.5 mm, respectively. *A. niger* was the most sensitive test organism at 12.5 µg/mL, with a zone of 14.75 mm produced, which further increased to 19 mm with a 2-fold increase in concentration to 25 µg/mL. Caspofungin was ineffective against *B. pseudobassiana* at concentrations below 150 µg/mL, with no zone produced at concentrations up to 12.5 µg/mL and a marginal zone of 8 mm produced at 25 µg/mL. A concentration of 150 µg/mL provided the greatest level of inhibition for all susceptible species, with significant zones ≥ 20 mm achieved. At this concentration, the order of sensitivity (from highest to lowest) is *B. pseudobassiana* (zone of 24.25 mm), *A. niger* (zone of 21.25 mm), *W. anomalus* (zone of 21 mm), *C. famata* (a) (zone of 21 mm), and *C famata* (b) (zone of 20 mm). A further increase in concentration to 200 µg/mL resulted in no significant variations in zone diameter. In addition to, being resistant to AMP B, both *M. pachydermatis* isolates further displayed resistance to caspofungin, along with *C. parapsilosis* and *C. neoformans* (data not shown), where *Cryptococcus* species are intrinsically resistant to the echinocandin antifungal drug. Results indicate that for phendione (Figure 1d), a 10 mM (2 × 10^3^ µg/mL) concentration was required to produce zones of cell death comparable to the antifungal agents. Indeed, at 10 mM phendione provided a zone diameter of 21 mm for *W. anomalus*, which was identical to the zone observed for caspofungin at 150 µg/mL. *B. pseudobassiana* also showed comparable patterns at these same concentrations, only varying by 1 mm, with a zone diameter of 23.25 mm produced at 10 mM phendione and a zone of 24.25 mm produced at 150 µg/mL caspofungin. The *Aspergillus niger* isolate and both *Candida famata* (a) and (b) isolates also followed this same pattern, where phendione provided respective zones of 17.75, 20.5 and 24.25 mm at 10 mM. This was comparable to zones of inhibition achieved for caspofungin, where a zone diameter of 19 mm was observed for *A. niger* at 25 µg/mL and respective zones of 21.25 and 20 mm observed for *C. famata* (a) and (b) at 150 µg/mL. Phendione provided significantly greater zones of inhibition for all remaining test species at a concentration of 10 mM, with respective zones of 17, 20, 23.76, and 16.75 mm observed for *C. parapsilosis*, *C. neoformans* and *M. pachydermatis* isolates (a) and (b). It should also be noted that for some test species the lower concentrations of phendione proved just as effective as the antifungal agents. For example, phendione produced a zone of 7 mm at 0.1 mM (20 µg/mL), which was identical to the zone produced by fluconazole at 6.25 µg/mL for *C. neoformans*. Similar results were also seen for *C. parapsilosis*, with a zone of 8 mm produced at 0.5 mM (100 µg/mL), corresponding to zone diameters produced by fluconazole at 200 µg/mL and AMP B at 50 µg/mL. *Candida famata* species also produced comparable zone diameters for phendione and fluconazole, where equivalent zone diameters slightly greater than 7 mm were provided at concentrations of 0.5 and 1 mM (phen) and 12.5 µg/mL (fluconazole). In addition, there was a dose-dependent increase in cell death for all test species up to 50 mM, where the zones produced were considerably greater than those observed with the antifungal agents. Specifically, 50 mM provided respective zone diameters (from lowest to highest) of 24.75, 25.25, 28.5, 29.25, 30, 30.25, 31, 35, and 47.25 mm for *A niger*, *C. parapsilosis*, *C. neoformans*, *M. pachydermatis* (b), *W. anomalus*, *C. famata* (a) and (b), *M. pachydermatis* (a) and (b), and *B. pseudobassiana*, a significant increase for all test species. 

Zones of inhibition produced by phendione for bacterial co-isolates in canine dermal cases of infection (see cases 3, 4, 5 and 7) are shown in Table 2. Results indicate that significantly lower concentrations of this drug are required to inhibit bacterial species, where zones produced at the higher concentrations of 50 and 100 µg/mL coincide with those observed with the antibiotic agents, specifically the aminoglycosides, glycopeptide, macrolides and penicillins. Furthermore, the bacterial species followed the same pattern as that seen with the fungal species, where a dose-dependent increase in concentration resulted in an increase in cell death for all test species. The Gram-positive staphylococcal strains were the most sensitive to treatment, with as little as 5 µg/mL phendione providing zones of 12.8 and 11 mm for *S. aureus* and *S. hominis,* respectively. Indeed, in comparison to the other test species, zones of inhibition for these strains were significantly greater, where a 10-fold lower concentration was required to produce equivalent results, i.e., *S. aureus* produced a zone of 15 mm at 10 µg/mL corresponding to zones observed at 100 µg/mL for *P. mirabilis* (15.3 mm), *S. canis* (15.3 mm) and *E. hirae* (15 mm). Whereas *S. hominis* produced a zone of 12.4 mm at 10 µg/mL, being similar to that obtained for the *E. faecalis* (13.8 mm) isolate at 100 µg/mL. Moreover, zone diameters for *Staphylococci* increased accordingly with a dose-dependent increase in concentration, where maximal zones >24 mm were achieved for both species at 100 µg/mL. On the other hand, a minimum concentration of 10 µg/mL was required to produce zones for the remaining test species, where zone diameters were marginal, ranging between 7–8 mm. The *Enterococcus* and *Streptococcus* species produced more or less identical zones across the concentration gradient reinforcing the genetic relatedness of these strains (where the *Enterococcus* species were formerly classified as group D streptococci before being established as a separate genus). Another pattern of sensitivity was seen among the Gram-negative strains, where mirroring zones of 12.8 mm were produced at 50 µg/mL, which further increased to 15.3 mm for *P. mirabilis* and 16.8 mm for *E. asburiae* with a 2-fold increase in concentration to 100 µg/mL. Additionally, all species were susceptible to phendione in the microdilution MIC assay, where the *S. aureus* isolate proved to once again be the most sensitive, with a MIC of 0.78 µg/mL. The next most sensitive test species were *S. hominis*, *S. canis* and *E. asburiae*, where a MIC value of 1.56 µg/mL was obtained, respectively. A 2-fold higher concentration of 3.13 µg/mL was required for *P. mirabilis* and *E. hirae* isolates, where the *E. faecalis* species was the most resistant to phendione treatment, with a MIC of 6.25 µg/mL. 

The minimum inhibitory concentrations obtained for the three antifungal agents and test agent phendione against the fungal isolates are presented in Table 3. Results show that all isolates displayed high levels of resistance to the three main families of clinically used antifungals, where the azole was found to be the overall most effective drug, with six of the nine isolates being sensitive. Though for many of these strains the MIC value was above the described clinical breakpoints (CBPs) defined by the EUCAST, with *C. famata* (a) the only test species to be deemed susceptible (S) where a result of 2 µg/mL falls just on the breakpoint border. The two remaining *Candida* species, *C. parapsilosis* and *C. famata* (b), were classed as resistant where MICs of 8 µg/mL were above the cut off value of 4 µg/mL for fluconazole. *Malassezia* strains (a) and (b) had respective MICs of 8 and 12 µg/mL, where there are no standardised breakpoints set for these species for the azoles or indeed any therapeutic agent. While species-specific CBPs do exist for some antifungal agents for *C. neoformans*, none have been implemented for fluconazole, where the breakpoints used for *Candida* species have generally been extrapolated to *C. neoformans*. Fluconazole provided an MIC value of 6 µg/mL for the *Cryptococcus* species, where in comparison to CBPs set for Candida species, it is evident elevated levels of this dug was required for complete inhibition. *W. anomalus*, *A. niger* and *B. pseudobassiana* all showed resistance to the azole agent, where no MIC was achieved across a concentration gradient of 0.016–256 µg/mL. However, more success was seen for the *W. anomalus* and *A. niger* isolates with the echinocandin class, where an MIC of 1.5 µg/mL was obtained, respectively, for caspofungin. The *C. famata* (b) isolate was also susceptible to caspofungin, with an MIC of 0.5 µg/mL, where micafungin (S ≤ 2 µg/mL; R > 2 µg/mL) and anidulafungin (S ≤ 2 µg/mL; R > 4 µg/mL) are used as markers for *caspofungin* susceptibility for Candida species (with no specific CBPs for caspofungin at present). The more resilient *C. famata* (a), *C. parapsilosis*, *Malassezia* and *B. pseudobassiana* strains were all resistant to caspofungin treatment, where *C. neoformans* is naturally resistant to this drug class. *A. niger* was found to be susceptible to AMP B treatment when applying the EUCAST CBPs, where a MIC value of 0.19 µg/mL falls within the CBP of 0.5 µg/mL. AMP B was also successful against the *W. anomalus* species, with a MIC of 1 µg/mL, where no current CBPs exist for this pathogen with respect to any antifungal agent. The *C. parapsilosis* species was the only other remaining test strain to show sensitivity to AMP B, with a MIC of 4 µg/mL. Though, this was notably above the CBP of 1 µg/mL, with the isolate deemed as resistant. When applying the epidemiological cut-off values (ECOFFs), almost all the isolates had non-wild-type phenotype drug resistance to fluconazole, AMP B and caspofungin. Only one isolate, *A. niger* exhibited a wild-type phenotype with respect to susceptibility to AMP B. In addition, *C. famata* (b) exhibited wild-type phenotype susceptibility to both fluconazole and caspofungin, where ECOFFs are 2 µg/mL, respectively. The *C. neoformans* species also fell within the ECOFF (16/32 µg/mL) for fluconazole. Phendione on the other hand proved to be a more successful treatment option, providing complete inhibition, where MICs were achieved for all test species. Moreover, the strains thar exhibited the greatest levels of resistance towards the antifungal agents, were found to be some of the most sensitive species to phendione, where MICs of 31.25 µg/mL and 250 µg/mL were obtained, respectively, for *Malassezia* species and *B pseudobassiana*. A MIC of 250 µg/mL was also maintained for *C. neoformans*, where the *Aspergillus niger* mould was also exceedingly susceptible to phendione, with a MIC of 62.5 µg/mL. The *Candidas’* were among the most resistant species tested, where both *C. famata* isolates had a MIC of 500 µg/mL, with a twofold higher concentration required for complete inhibition of *C. parapsilosis*. A concentration of 1 × 10^3^ µg/mL phendione was also requited for complete inhibition of *W. anomalus* species. 

Alphabetical lettering A through to Z indicates significance in zone diameter at *p* < 0.05 where sensitivity to test chemicals for this species varied significantly. The results of the biocidal testing of novel agents peracetic acid (PA) and triameen are summarised in Table 4, where test agents provided significantly greater zones than the alcohol-based control agent (IP70). Findings show that both PA and triameen were effective against *W. anomalus*, *C. neoformans*, *Candida* and *Malassezia*, where zone diameters increased in a dose-dependent fashion. Triameen appeared to generate greater effects for these organisms at the lower concentrations of 0.01 and 0.1% in comparison to PA, particularly for *C. neoformans* isolate, where zones produced at 0.1% were significantly greater (>10 mm). However, a concentration of 1% PA provided greater zone diameters for *Candida* and *Malassezia* species, where triameen remained the disinfectant of choice for *W. anomalus* and *C. neoformans* at this concentration. On the other hand, the *A. niger* mould and entomopathogenic *B. pseudobassiana* strain proved to be more resistant to disinfectant treatment, where triameen provided no zone of inhibition for these species, with an exception at 1% for *A. niger*, where a marginal zone of 9.5 mm was observed. Albeit, a concentration of 1% PA did prove extremely successful against these two species, where zones of 18 and 35 mm were produced, respectively, for *A. niger* and *B. pseudobassiana*. Overall, the presence of BSA had no significant bearing on the test chemicals ability to inhibit these species. Although, for *W. anomalus*, the presence of BSA did influence zone diameter, where significant reductions for both PA (<5 mm) and Triameen (<6.5 mm) were observed at 10 g/L BSA. *M. pachydermatis* species were also affected by the presence of BSA, though in this case the presence of BSA continuously provided increased activity of test agents (where PA was more affected), suggesting that the presence of serum albumin somehow increased the cellular uptake of disinfectant by these species. 

## 3. Discussion 

Chronic dermatophytosis in dogs and cats represents an ongoing issue in daily veterinary practice, where the incidence of infections caused by yeast and mould species is becoming more frequent. Fungal mycoses by non-dermatophytes typically arise as a secondary problem in the animal, where predisposing factors such as poor nutrition, age, breed, or weakened immunity influence the host’s risk of infection. This unnecessary use of antibiotics not only worsens symptoms of mycoses but is also a major driver of AMR, where exposure allows bacterial pathogens to upgrade their arsenal of defence via transposable elements, plasmid acquisition and horizontal gene transfer. The production of βeta lactamase enzymes is one such defence mechanism that many bacteria have evolved over the years, being the most common mode of resistance to first line βeta lactam agents among Gram-negative organisms. The presence of AmpC βeta lactamase in recent years has further complicated treatment options, where these enzymes renounce resistance to all βeta lactamase inhibitors, such as clavulanate. It is important to note that the patient harbouring this species (see case description for patient 3) was initially prescribed amox clav, being suspected of bacterial pyoderma, where amox clav is among the many recognised portent inducers of AmpC overexpression. As demonstrated by this study, for all cases, the causative agent was determined to be fungal, where all the aforementioned predisposing factors evidently complicated diagnosis and disease aetiology, with the presence of a fungal causative agent initially overlooked in many cases. Antifungal resistance is another growing dynamic that has been paid inadequate attention until recent years, where increasing evidence proposes a post antifungal era may also be on the horizon. Certainly, the findings of this study reiterate this claim, with test species showing resistance to at least one antifungal agent, with several species exhibiting MDR or pan-drug resistance (PDR). In the Kirby–Bauer assay, caspofungin exerted the greatest level of inhibition for susceptible species, followed by fluconazole and AMP B, though this unanimous order was not individually adhered to among the test strains. Additionally, many species showed high levels of resistance even with increasing concentrations of drug, particularly the *Malassezia* and *C. parapsilosis* test strains. In the context of susceptibility testing however the MIC is considered the gold standard in determining the success of a therapeutic agent, where the EUCAST have established clinical breakpoints and ECOFFs to aid clinicians in selecting the best agent for a microbial disease [3]. Though these are limited to the more common *Candida*, *Cryptococcal*, *Aspergillus* and *Trichophyton* species, with studies herein, highlighting the need for defined species-specific breakpoints, particularly for *Malassezia* and *W. anomalus* species. Moreover, studies reiterate the need for specific animal breakpoints, where CBPs used in this study were extrapolated from EUCAST guidelines for human fungal infections. In the MIC assay, fluconazole exerted the greatest effects, being active against six of the nine test species, with caspofungin and AMP B only providing MICs for three strains. Fluconazole is used to treat many local and systemic infections in humans, such mucosal candidiasis, invasive candidiasis, cryptococcal meningitis and invasive aspergillosis. Additionally, this drug is often administered to patients in ICU on a prophylactic basis to reduce the risk of invasive infection, particularly candidemia (where a dose of 200–400 mg/day is *recommended*). In this study, only one isolate, *C. famata* (a), was deemed susceptible to the azole agent, where elevated MICs above described EUCAST CBPs were observed for *C. famata* (b), *C. parapsilosis* and *M. pachydermatis* species (respective MICs of 8 and 12 µg/mL) and *C. neoformans* (6 µg/mL). However, finding of an elevated MIC for an antifungal drug should not necessarily preclude its use, especially if the use of alternative antifungal therapies for the patient has proved ineffective, which was the case for *C. famata* (b), *C. parapsilosis*, *C. neoformans* and *M. pachydermatis* isolates. AMP B is licensed for treatment of systemic or severe *Candida*, *Aspergillus* and *Cryptococcus* infections in humans (among other fungal infections), being administered IV at a dose of 1 mg/10 mL or 0.1 mg/mL. AMP B proved to be a successful option for the fluconazole-resistant *A. niger* and *W. anomalus* strains, where MICs were 0.19 (CBP = 0.5 µg/mL) and 1 µg/mL, respectively. *C. parapsilosis* was the only remaining species to show sensitivity to this drug, but an MIC of 4 µg/mL was above the EUCAST CBP (1 µg/mL). Increasing concentrations of AMP B are generally not feasible, with its use accompanied by dose-limited toxicities, where nephrotoxicity is the most serious. Indeed, recent revision of the EUCAST susceptibility categorisation (S, I, R) guidelines precludes the I category for AMP B, where this is now only applied in situations where there is a high likelihood of therapeutic success because exposure to the agent is increased by adjusting the dosing regimen or by its concentration at the site of infection [3]. However, as aforementioned, the MIC values do not always directly associate with response to antifungal treatment, where combination therapy with azoles is often considered [16]. Certainly, this combination route my prove effective in the case of the caspofungin-resistant *C. parapsilosis*, where this strain showed reduced susceptibility to both AMP B and fluconazole. Caspofungin belongs to the most recently developed class of antifungals, the echinocandins, being administered as an intravenous treatment at dose of 70 mg/per day in humans [17]. Depending on the genera of fungi, these compounds exert either fungicidal (dimorphic yeasts) or fungistatic effects (moulds), where they are ineffective in infections caused by Cryptococci. Overall caspofungin treatment was unsuccessful, where *A. niger*, *C. famata* (a) and *W. anomalus* were the only species to show wild-type phenotype susceptibly to this drug. These findings coincide with recent reports in the literature, where numerous studies describe increased MICs and acquired resistance to several different drug classes [18]. Findings further corroborate global reports showing that there has been a gradual shift in epidemiology from *Candida albicans* to *Non-Candida albican* strains, where species such as *C. parapsilosis C. auris*, *C. glabrata* and *C. famata* are becoming more frequent causes of disease [19]. Initially, this variation in epidemiology was thought to be a result of the widespread use of the azoles, particularly as prophylactic agents. However, this temporal viewpoint has not been consistently demonstrated, with other factors including exposure to antibiotics, immunosuppressive therapy, and predisposing medical conditions of the patient a more likely predicator of the infection distribution [20]. Such factors would also account for the increasing appearance of extremely rare and often lethal fungal pathogens (such as *W. anomalus* and *B. pseudobassiana*) and may further relate to resistance levels seen in this study, where the presence of these xenobiotic compounds have been reported to activate efflux pumps (a non-selective resistance mechanism) in *Candida*, *Cryptococcus* and *Aspergillus* species. Furthermore, the adaptive nature of fungi has enabled them to develop multiple defences mechanisms to resist antifungal therapy, where they typically occur collectively, and include drug target alteration or overexpression, reduction in intracellular accumulation of the drug (pump overexpression), or activation of cellular stress response or tolerance pathways [21]. Variation in drug resistance can be observed at multiple levels. For example, azole resistance in *Candida* species is generally by overexpression of drug efflux pumps and modification of the drug target (ERG11), whereas, in *Aspergillus* species resistance is primarily caused by target modification (Cyp51A). Variations also exist between strains within a species. For instance, *C. krusei* and *C. glabrata* both possess intrinsic resistance to fluconazole compared to other *Candida* species. Additionally, fungi possess a number of facilitating attributes such as dimorphism, adhesins and invasins, proteases and biofilm-forming capabilities [22], which can further enhance pathogenesis and lead to infectious species crossing over from the dermis to the systemic circulation causing invasive mycoses. Opportunistic yeasts such as *M. pachydermatis*, *W. anomalus* and *B. pseudobassiana* are becoming of increasing importance in both animal and human diseases, where these pathogens are now being reported in cases of fungemia, where neonates and ICU patients are at most risk [5,23,24,25,26,27]. Such findings emphasise the importance of considering these pathogens within the diagnostics realm, where current CBPs are lacking. The zoonotic potential of these resistant organisms further poses a threat to human health, where increasing reports of *M. pachydermatis* in humans is thought to be attributed to the emergence of this species in canine dermatitis and otitis [24,28], where zooanthroponosis (reverse zoonosis) also poses a risk to animals. To overcome rising resistance, novel alternative therapies to replace or enhance antifungal agents are required, where phendione offers a potential option for treatment of such aggressive isolates. Phendione provided complete inhibition of fungal growth for all test species, where the *Malassezia* strains were the most sensitive with MICs as low as 0.156 mM (or 62.5 µg/mL). On the other hand, *C. parapsilosis* and *W. anomalus* were the most resistant species to treatment, where respective MICs were 5 mM. Previous studies conducted by Meade et al., 2019 also report MICs of 5 mM for *Candida albican* and *Candida krusei* isolates, where in comparison *C. famata* proved slightly more susceptible with a 2-fold lower concentration of 2.5 mM inhibiting fungal growth. Research to date shows that this lipophilic drug exerts its antifungal effects by damaging mitochondrial function, uncoupling respiration, causing non-specific DNA cleavage, disrupting cell division and inducing gross distortions in fungal cell morphology [14]. Whereas the potency of phendione against bacterial species is associated with its avid cheating properties, having the ability to selectively disturb essentials metals necessary for bacterial physiology and metabolism. Studies find phendione was even more successful against all co-isolated bacterial species (Table 2), where MICs were ≤3.13 µg/mL, demonstrating both aggressive antifungal and antibacterial activity of this agent against veterinary isolates. In this context, it is extremely relevant to highlight the usefulness of phendione in treating *Malassezia pachydermatis* fungal infections, where a significant number of dogs affected with *Malassezia* dermatitis are often concurrently afflicted with bacteria Staphylococcal pyoderma [29]. Indeed, in this study both reported cases of *M. pachydermatis* also harboured co-isolated Staphylococcal species, where a symbiotic relationship is thought to exist between these two organisms. Moreover, phendione proves promising for treatment of spore forming pathogens, being highly active against the fluconazole-resistant *A. niger* mould and the PDR *B. pseudobassiana*. However, further clinical in vitro studies such as drug biocompatibility and pharmacokinetics, among others, are required to complement microbiological findings. Whilst offering alternative therapeutics is one way of dealing with antimicrobial resistance, a more useful method to combat the issue is working towards infection prevention. Effective biocidal options play a crucial role in reducing drug-resistant fungi and their spore and/or biofilm counterparts that persist on clinical surfaces and the surrounding environment, where nosocomial infections represent an ongoing threat to animals. In this study, both peracetic acid and triameen test agents demonstrated excellent activity against resistant *Candida*, *Malassezia*, *W. anomalus* and *C. neoformans* species. On the other hand, triameen failed to inhibit the growth of *Aspergillus niger* and *B. pseudobassiana* isolates, where the peracetic acid-based disinfectant proved superior for these species. 

## 4. Methodology

Amphotericin B (AMP B), fluconazole and caspofungin were sourced from Sigma Aldrich, Dublin, Ireland, and 1, 10-phenanthroline (phendione) was sourced from VWR, Ireland. All therapeutic agents were made to stock solutions by dissolving the drug powder in adequate volumes of dimethyl sulfoxide (DMSO) (*w/v*). Once completely dissolved with no precipitation evident, working concentrations of all test agents where then prepared based on EUCAST recommendations (2020). For each test method, the effect of DMSO on fungal growth was also evaluated by testing the different concentrations without the antifungal agent to negate the effect of DMSO induced toxicity, as it was used for drug dissolution. In addition, gradient concentration strips (Liofilchem^®^ MIC test strip (MTS), launch diagnostics, Ireland) for all the above-mentioned antifungal agents were used. 

### 4.1. Animal Morbidity and Clinical Symptoms 

Canine patient 1: A 3-year-old male rough coated collie presenting with a week-long history of pruritis and foul-smelling coat. On examination, there was a clear track along dorsal midline of his neck from the base of the skull to the withers (shoulder blade level). There was a thin purulent discharge with a musty small. There was bilateral erythema surrounding the lesion. The entire area was highly pruritic. Patient was treated biweekly with ketoconazole and chlorhexidine wash and oral clindamycin at a dose of 5.5 mg BID for 2 weeks. Skin scrape using a blunted scalpel blade and microbial culture identified two fungal species, *Candida famata* and *Wickerhamomyces anomalus*, via selective agars and PCR identification. Diagnosis of invasive cutaneous dermal infection with the zoonotic *Candida famata* and *Wickerhamomyces anomalus* as the causative agents. 

Canine patient 2: A 6-year-old wheatador with atopy diagnosed 5 years ago. Allergy testing showed multiple food allergies and grass pollen and UK weed allergy; had been on a low dose of prednisolone treatment for 5 years to manage symptoms of atopy (0.2 mg per kg BID). Dog presented with non-pruritic, crusty rash on chest. Prednisolone treatment was temporarily stopped (3 weeks and patient was treated biweekly with ketoconazole and chlorhexidine wash). Skin scrape, swabs and microbial culture identified *Candida famata* via selective agars and PCR identification. Diagnosis of cutaneous candidiasis with the zoonotic *Candida famata* as the causative agent. 

Canine patient 3: A 5-year-old neutered male. Presented with lameness, pruritis and biting at left front paw and carpus. Erythema and clear productive folliculitis. Suspected bacterial dermatitis. Treated with chlorhexidine wash once daily and Amoxicillin with Clavulanic Acid at a rate of 1.25 mg per kg twice daily x 20 days. Skin scrape and microbial culture identified the lipophilic *Malassezia pachydermatis* yeast and three bacterial species, *Staphylococcus hominis*, *Enterococcus faecium and Enterobacter asburiae* via selective agars and PCR identification. Reoccurring symptoms post antibiotic treatment established a diagnosis of *Malassezia* dermatitis. 

Canine patient 4: A 14-year-old, male, intact border collie, presenting with generalised pruritis, painful dermatitis under left ear, along ventral abdomen and around anus, with anal furunculosis causing extreme discomfort and biting at rear end. Left ear was producing a thick purulent discharge. The peri anal region was showing signs of dehiscence and producing a haemopurulent discharge. The ventral abdomen was not productive but warm to touch. Due to patient’s age, chronic problem with severe osteoarthritis and inability to wash him at home (developed aggressive behaviour due to painful lesions) the owner opted to have dog euthanised on welfare grounds. Skin scrape, swabs and microbial culture identified *Malassezia pachydermatis*, and co-isolated bacterial species, *Staphylococcus* sp. and *Streptococcus canis* via selective agars and PCR identification. Diagnosis of otomycosis due to *Malassezia pachydermatis*, where concurrent bacterial infections are often associated. 

Canine patient 5: A 5-year-old, intact male Scottish terrier. Presented with unilateral pruritis associated with a discharging right ear canal. ‘Musty’ smell evident from ear. Painful to touch. Ear was cleaned with an alcohol-containing solution and treated twice daily with fucidic acid, framomycetin, nyastatin and prednisolone containing ear drops. Skin scrape and microbial culture identified *Aspergillus niger* and *Enterococcus hirae* species. Diagnosis of Otomycosis with the zoonotic *Aspergillus niger* as the causative agent. 

Canine patient 6: An 8-year-old neutered male Labrador with atopic dermatitis and food allergies. Poor owner compliance meant patient regularly reverted to allergen-containing diets, giving chronic irritated skin and allowing recurrent secondary infections. On this occasion, the patient showed mild pruritis but had a profound musty smell from coat, greasy skin and scaley skin (dry flakes caught in coat). Patient was treated with a hypoallergenic prescription diet × 6 weeks to control atopy, biweekly chlorhexidine and ketoconazole wash × 6 weeks, Oral meloxicam at a rate of 0.1 mg/kg once daily orally and oral clindamycin at a rate of 5.5 mg per kg BID × 20 days. Dog was rehomed as the owner could not afford ongoing management costs and with controlled diet, no longer shows recurrences. Skin scrape and microbial culture identified *Cryptococcus neoformans* via selective agars and PCR identification. Diagnosis of cutaneous cryptococcosis with the zoonotic *Cryptococcus neoformans* as the causative agent. 

Canine patient 7: Patient presented with ongoing dermatitis that was originally diagnosed as bacterial pyoderma, showing no signs of improvement with antibiotic treatment. Upon further examination, patient was suspected of ringworm infection, where several patches of alopecia, erythema and folliculitis were clearly visible. Fungal culture of hairs and scrapings from afflicted areas identified the Entomopathogenic *Beauveria pseudobassiana fungus* as the causative agent of disease via morphological characteristics (pure white powdery appearance on sabouraud agar), microscopic analysis and PCR. Co-isolated bacterial species included *Proteus mirabilis* where antibiotic susceptibility testing (Table 1) showed no pattern of resistance. 

Feline patient 1: A 2-year-old female neutered domestic shorthaired cat. Patient presented with lethargy, inappetence, poor coat quality (thin coat covering body, thick scaley skin, dry flakes of skin throughout coat) and mildly pruritic, showing discomfort when stroked. Patient weighed 1.4 kg and was markedly undersized for her age and breed. Daily oral itraconazole at a dose of 5 mg/kg on alternating weeks and biweekly chlorhexidine/ketoconazole washing was planned. Initial response to treatment was promising for skin and coat (more comfortable when skin touched and pruritis resolved). In the interim, a positive result of feline infectious peritonitis (FIP—caused by feline corona virus) was obtained and patient was euthanised on welfare grounds. Skin scrape, swabs and microbial analysis identified *Candida parapsilosis* as the causative agent via selective agars and PCR identification. 

### 4.2. Fungal Isolation, Identification, Culture and Maintenance 

Collected samples of infection (skin scrapes) were inoculated in sabouraud dextrose broth (Cruinn Diagnostics, Dublin, Ireland) and incubated under rotary conditions (125 rpm) at varying temperatures of 25, 30 and 37 °C, respectively, for up to 10 days, streaking intermittently onto sabouraud dextrose agar (Cruinn Diagnostics, Dublin, Ireland). Individual colonies were re-streaked for isolation and pure isolated colonies inoculated into sabouraud broth for further biochemical characterisation. Colonies were identified based on their morphological characteristics, biochemical profile and growth on selective agars, specifically HiMedia™ Cryptococcus Differential Agar, CHROMagar™ Candida and Malassezia (CHROMagar, Paris, France). Identity was confirmed via colony polymerase chain reaction (PCR). For all test isolates, the direct colony PCR method was performed, where 1 μL of fungal colony suspension was used directly as the DNA template. For *Candida* isolates, *C. neoformans* and *W. anomalus* a single colony was picked from a 48 h culture using a sterile micropipette tip and suspended in 100 µL sterile deionised water. A 1 µL suspension was directly used in PCR reaction. For *M. pachydermatis*, *A. niger* and *B. pseudobassiana*, fungal cells of 7-day-old cultures were gently scraped and suspended in 400 µL of deionised water. Sterile glass beads (400–600 µm) were then added to the 400 µL suspension, and the mixture vortexed using a Minilys homogeniser (Bertin Instruments, VWR) at a speed of 5000 rpm for 20 s in order to lyse cells. After resting shortly, 1 µL of supernatant was used as the template. Fungal primers ITS1-F 5′-CTT GGT CAT TTA GAG GAA GTA A-3′ and ITS4 5′-TCCTCCGCTTATTGATATGC-3′ (Sigma Aldrich, Dublin, Ireland) were used for direct amplification of intergenic spacer regions (ITS) of rDNA. Direct colony PCR was performed in a total reaction volume of 20 µL, containing 17 µL red Taq 1.1x master mix (VWR, Dublin, Ireland), 1 µL ITS1F, 1 µL ITS4 and 1 µL of selected colony suspension. DNA amplification was performed in a thermo cycler (VWR, Dublin, Ireland) using the recommended parameters. Following DNA amplification, the PCR products were examined via electrophoresis on a 1% *w/v* agarose gel run at 110 volts for 45 min. Successful reactions were sent to GATC (Eurofins, UK) for clean up and gene sequencing of products. Strains were stored long term in 20% glycerol at −20 °C and short term at 4 °C in sabouraud dextrose broth/agar, where supplementation of sabouraud dextrose agar with 1% tween 80 and 2% olive oil was required for *M. pachydermatis*. Identity was confirmed via Gram stain and selective agars prior to each experimental set up. 

### 4.3. Inoculum Preparation

Stock inoculum suspensions of *Candida* sp., *C. neoformans* and *W. anomalus* were prepared by suspending colonies obtained from respective 48 h and 72 h cultures grown on sabouraud dextrose agar in sterile distilled water and vertexing for 10 s. In the same way, suspensions of *M. pachydermatis* were prepared from fresh 5-day-old cultures grown on sabouraud dextrose agar supplemented with 1% tween and 2% olive oil and incubated at 35 °C. However, due to the tendency of *M. pachydermatis* cells to readily form clumps, owing to the butyrous nature of colonies, the suspensions were subsequently vortexed with glass beads at approximately 3000 rpm for 20 s in order to form a homogeneous suspension. A. niger and *B. pseudobassiana* spore inoculums were prepared by subculturing on sabouraud dextrose agar and incubating for up to 5 days at 35 °C and for up to 14 days at 25 °C, respectively, to allow for sporulation. Conidial suspensions were then prepared by flooding the cultures with 5 mL of sterile water supplemented with 0.1% Tween 20 as a wetting agent and gently scraping the surface with a sterile spreader. Resulting suspensions were subsequently filtered using sterile whatman filter paper (Cruinn Diagnostics, Dublin, Ireland) with a pore size of 11 µm to remove rough parts of medium and mycelia, and viability determined by standard plate count method. All test suspensions were adjusted accordingly to comply with EUCAST guidelines for each test method. 

### 4.4. Kirby–Bauer 

Antifungal susceptibility patterns of isolates to Amp B, caspofungin, fluconazole (Sigma Aldrich, Dublin, Ireland) and phendione were assessed via disc diffusion method. The Kirby–Bauer assay was conducted as per Meade et el., 2019 with zones of inhibition measured in millimetre (mm) following 24 h incubation for *A. niger* at 37 °C, 48 h incubation for *Candida* sp. and *C. neoformans at* 30 °C, 72 h incubation for *M. pachydermatis* and W. *anomalus* at 35 and 25 °C, respectively, and 5 days incubation for *B. psuedobassiana* at 26 °C. Concentrations tested ranged from 2.5 to 50 µg/mL for Amp B, 2.5 to 150 µg/mL for caspofungin, 2.5 to 250 µg/mL for fluconazole and 0.1 to 50 mM for phendione. The Kirby–Bauer assay was also carried on co-isolated bacterial species using Muller–Hinton agar (Cruinn Diagnostics, Dublin, Ireland) and a range of antibiotic susceptibility disks (Fisher scientific, Ireland), where test agent phendione was also accessed. 

### 4.5. Minimum Inhibitory Assay 

MIC determination for phendione was performed in flat bottom 96 well plates using RMPI 1640 media (with L -glutamine, with pH indicator, but without bicarbonate) (Fisher scientific, Ireland), supplemented with 2% Glucose and 3-(N-morpholino) propanesulfonic acid (MOPS) (VWR, Ireland) at a final concentration of 0.165 mol/L, pH 7.0. Testing was conducted according to the EUCAST guidelines where final well concentrations ranged from 10 to 0.019 mM. Briefly, phendione test solutions were prepared at 2× the final concentration in double strength RPMI media and 100 µL added to the respective well of the 96-well microtiter plates, being arranged in twofold dilution from left to right and in triplicate. Control wells contained 100 µL sterile drug-free medium. Inoculums prepared as previously described were adjusted to 2.5 × 10^5^ CFU/mL in sterile water. Wells were then inoculated with 100 µL of the respective test suspension, including growth control wells with the final column of the plate remaining blank (containing just media and water). Plates were incubated without agitation for 24–48 h at 30 °C for *Candida* sp. and *C. neoformans*, 37 °C for *A. niger*, 25 °C *W. anomalus* and 26 °C for *B. pseudobassiana* for up to 72 h. MICs were measured visually and spectrophotometrically at 530 nm. The value of the blank (background) was subtracted from readings for the rest of the wells. As there are no standardised reference methods specifically dedicated for antifungal susceptibility testing of *Malassezia* yeast, and conditions employed by the EUCAST methods are universally accepted to be not suitable for testing of Malassezia species (due to their lipophilicity), an alternative urea broth microdilution method was employed. In this method, Christensen’s urea broth (Cruinn Diagnostics, Ireland) modified to grow these lipophilic yeast-like fungi via the addition of Tween 40 (0.5%) and Tween 80 (0.1%) was used. The urease broth method is a colorimetry based assay whereby the incorporated urea in the test media is converted to ammonia by urease activity of the yeast, resulting in an alkaline change in pH change (from yellow to pink) which is proportional to the number of surviving yeast. Microtiter plates were prepared in the same manner as previously described, with wells containing 100 µL of 2× the final drug concentration but in Christensen urea broth. The microdilution plates were then inoculated with 100μL of 2.5 × 10^5^ CFU/mL yeast suspension, giving a final inoculum density of ca 1.25 × 10^5^ CFU/mL. MICs were measured visually and spectrophotometrically at 545 nm. 

MICs for amphotericin B, fluconazole and caspofungin were determined by gradient concentration strip method (Liofilchem^®^ MTS, Launch diagnostics, Ireland). In this technique, a predefined exponential gradient of antifungal drug is impregnated on a paper strip across 15 two-fold dilutions like those of a conventional MIC method. A homogenous suspension of each test strain was prepared as previously described and adjusted to final cell densities of 0.5 McFarland (or ca. 1 × 10^7^ cells/mL). RPMI 1640 agar plates supplemented with MOPS (0.165 M) and 2% glucose (Liofilchem^®^, Launch Diagnostics, Ireland) were inoculated with the suspension by introducing a sterile swab into the culture and streaking across the surface. The procedure was repeated two more times rotating the plate approximately 60 degrees each time to ensure an even distribution of inoculum. Once excess moisture was absorbed and plates were completely dry, a test strip of the respective antifungal agent was applied to the surface of the agar. Plates were inverted and incubated accordingly for each test species. MICs were read visually where the inhibition ellipse intersects the MIC scale of the strip, where overgrowth by filaments bending into the ellipse was ignored. Results were then compared to standardised EUCAT clinical breakpoints, where species were classed as resistant (R), susceptible (S) or susceptible, increased exposure (I). 

The MIC assay was also conducted for all co-isolated bacterial species for phendione using Muller–Hinton broth, where standard EUCAST test conditions were implied. 

### 4.6. Novel Biocidal Options

The antimicrobial agents investigated in this study are pure biocides used as disinfectants alone or in commercial brands. The concentrations described are the concentration of the active component and include the concentration used following manufactures instructions. Test solutions studied for use in veterinary areas and as farm disinfectants utilise peracetic acid or triameen (*N*-(3-aminopropyl)-*N*-dodecylpropane-1,3- diamine) as active ingredients. Peracetic acid is a powerful oxidant capable of oxidising the outer cell membranes of micro-organisms, acting as a biocidal disinfectant. Triameen is a fatty amine derivative and a highly effective antimicrobial agent. Studies by Meade et al. have demonstrated the antimicrobial potential of both disinfectants on reference strains sourced from the American Type Culture Collection (ATCC) bank. In the present study, the activity of both biocides will be determined against multidrug-resistant fungal pathogens isolated from incidents of dermatomycosis. 

#### Kirby–Bauer Disk Diffusion Assay Using Novel Biocidal Solutions 

The Kirby–Bauer assay was carried out on all test isolates to determine the effect of disinfectants on microbial species with the presence and absence of an interfering substance. 1 mL of ca. 1 × 10^7^ microbial cells was added to 9 mL BSA to give a working microbial count of 10^6^ cells in solution with 3 g/L or with 10 g/L BSA and 10 g/L yeast extract (YE) (Sigma, Ireland) as low- and high-level interfering substance. Subsequently, 100μL 10⁶ cells/mL microbial suspension were transferred onto replicate agar plates and spread with a sterile L-shaped spreader (Cruinn Diagnostics) to ensure even disruption across the agar surface. Filter disks (6 mm) were immersed in the test biocide solutions at concentrations of 0.01, 0.1 and 1% (*v/v*) for 15 s and excess solution was allowed to drip off the disk. Subsequently, the disk was placed on the inoculated plate. Plates were then incubated for the required time period and temperature. Zones of inhibition were then measured using a Vernier calliper in millimetres as per Meade et al., for each test chemical and each test organism. 

### 4.7. Statistics

All the experiments were performed three times with three plate replicates for each experimental data point, providing a mean result for each test species and antifungal susceptibility (±standard deviation). The log_10_ inhibition of growth was calculated as the log_10_ of the ratio of the concentration (cfu/mL) of the non-treated (N0) and treated (N) samples [log_10_ (N0/N)]. Student’s T tests were conducted to determine significance levels (*p* < 0.05) of bacterial susceptibility to treatment using Minitab 16 (Minitab Ltd., Coventry, UK). 

## 5. Conclusions 

The phenomenon of immunosuppression (pathological or iatrogenic) has been recognised as a major contributor of increasing fungal disease, where more and more patients with chronic immunosuppressing conditions require antifungal treatment as part of their treatment plan. This rising rate of infection has been accompanied by increasing levels of antifungal resistance, where fungal pathogens are following the same pattern of exposure-resistance as seen with bacteria in the imminent antibiotic crisis. In order to defeat AMR, accurate and timely diagnosis is of utmost importance, where selection of fungal therapy must now also consider the shift in predominance from *C. albican* to *non-albican* species, as well as rarer (and often more fatal) *non-Candida* species. This study presents eight examples of specific clinical cases that required greater application of existing fungal diagnostics and improved overall fungal diagnostic capability, where CBPs were lacking for many fungal pathogens. The presence of resistant species in companion animals such as those described herein, represents an often-unrecognised route of disease transmission, where zoonosis poses a significant risk. The development of new treatment options is, therefore, essential for both animal and human medicine, where test agent phendione showed promising potential as both an antifungal and antibacterial agent. Additionally, test biocidal options triameen and peracetic acid both showed high levels of antifungal activity. In particular, the peracetic acid-based disinfectant shows promise for controlling the spread of microbial species in veterinary settings, also being active against spore formers. 

## Figures and Tables

**Table 1:** Antibiotic profile for bacterial co-isolates in companion animal dermal cases of infection to a range of antibiotics from varying drug classes, as determined via measurement of zones of inhibition (mm).

Drug Class	Aminoglycoside	Glycopeptide	Macrolide		Penicillin		Penicillin-Like	Cephalosporins			Carbapenems			Quinolones		Tetracycline
Antibiotic	Streptomycin	Vancomycin	Azithromycin	Erythromycin	Penicillin	Ampicillin	Amox/clav	Cefpodoxime	Cefotaxime	Ceftriaxone	Doripenem	Meropenem	Imipenem	Ciprofloxacin	Levofloxacin	Doxycycline
Conc. µg/disc	10	30	15	15	10	10	20:10	10	5	30	10	10	10	5	5	30
*P. mirabilis*	16	R	10	R	16	17	20	28	28	33	21	25	20	31	33	R
*Staphylococcus* sp.	20	13	25	30	20	19	23	34	29	33	33	28	45	30	31	31
*Streptococcus canis*	15	26	25	24	49	32	40	33	30	35	30	30	45	21	25	28
*Enterobacter asburiae*	17	R	10	R	R	R	11	26	27	27	25	27	30	33	29	19
*Staphylococcus hominis*	23	20	R	R	40	35	37	29	20	28	38	35	50	35	29	35
*E. faecium*	12	18	12	15	11	18	23	8	7	14	7	20	7	14	14	23
*E. hirae*	R	20	20	24	18	17	24	R	R	R	R	R	22	17	17	22

R donates complete resistance to antibiotic, evident by absence of any zone of inhibition.

**Table 2:** Zones of inhibition (mm) produced by phendione for bacterial co-isolates in canine dermal cases of infection and minimum inhibitory concentration.

Phendione (µg/mL)	Zone of Inhibition(m)						
	*P. mirabilis*	*S. aureus*	*S. canis*	*E. asburiae*	*S. hominis*	*E. faecium*	*E. hirae*
**5.00**	0.00	12.8(±0.3)C	0.00	0.00	11.0(±0.4)B	0.00	0.00
**10.00**	8.00(±0.4)A	15.0(±0.4)D	7.0(±0.4)E	8.0(±0.4)A	12.4(±0.2)C	7.3(±0.3)E	7.0(±0.4)E
**20.00**	10.8(±0.3)B	18.0(±0.3)F	8.3(±0.3)A	9.3(±0.4)I	17.6(±0.4)F	8.4(±0.3)A	8.3(±0.2)A
**50.00**	12.8(±0.4)C	21.3(±0.6)G	11.3(±0.5)B	12.8(±0.6)C	21.0(±0.2)G	12.5(±0.5)C	11.0(±0.5)B
**100.00**	15.3(±0.4)D	24.3(±0.4)H	15.3(±0.4)D	16.8(±0.6)J	24.8(±0.4)H	13.8(±0.4)K	15.0(±0.6)D
**MIC(µg/mL)**	3.13	0.78	1.56	1.56	1.56	6.25	3.13

Alphabetical letters A through to K denote significant difference at *p* < 0.05 where test species differ significantly in their susceptibility to the test agent.

**Table 3:** MIC results for fungal dermal isolates of companion animal disease for fluconazole, AMP B, caspofungin (based on gradient strip method) and phendione (based on microdilution).

Fungal Isolate	Fluconazole	Amp B	Caspofungin	Phendione
	µg/mL			µg/mL
*C. parapsilosis*	8 R	4 R	R	1000
*C. famata (a)*	8	R	R	500
*C. famata (b)*	2 S	R	0.5	500
*W. anomalus*	R	1	1.5	5
*C. neoformans*	6	R	R	250
*M. pachydermatis (a)*	12	R	R	31.25
*M. pachydermatis (b)*	8	R	R	31.25
*A. niger (bobby)*	R	0.19 S	1.5	62.5
*B. pseudobassiana*	R	R	R	250

Fluconazole (strip range = 0.016–256 µg/mL); AMP B and Caspofungin (strip range = 0.002–32 µg/mL). R: resistant and S: susceptible to the test agent. Clinical breakpoints of 2 µg/mL for Candida for fluconazole and 1 for *C. neoformans*, 0.5 µg/mL for A. niger for AMP B apply. Breakpoints listed are those used in human diagnostics, where specific-animal breakpoints are lacking.

**Table 4:** Zones of inhibition (mm) produced by antimicrobial biocides against fungal isolates in the absence and presence of interfering substance BSA at 3 and 10 g/L (+10 g/L YE) (± standard deviation).

Fungal Isolate	BSA (g/L)	Biocide Test Substance						
		Peracetic Acid			Triameen			IPA
		0.01%	0.1%	1%	0.01%	0.1%	1%	70%
*C. parapsilosis*	*0*	7.0(±0.1)A	10.0(±0.4)E	25.0(±1.4)I	7.0(±0.1)A	11.8(±0.3)C	16.3(±0.4)N	11.5(±0.4)G
	*3*	7.0(±0.6)A	8.5(±0.2)B	22.5(±1.6)J	7.5(±0.2)A	11.8(±0.4)C	12.8(±0.5)H	11.5(±0.1)G
	10 *	7.0(±0.2)A	8.0(±1.1)B	20.8(±1.2)K	7.0(±0.1)A	11.0(±0.1)G	15.5(±0.2)N	11.5(±0.2)G
*C. famata (a)*	*0*	7.8 (±0.6)B	12.5(±0.7)C	26.8(±1.4)II	8.8(±0.3)F	18.5 (±1.0)X	25.5(±0.3)I	9.0(±0.4)Y
	*3*	12.0(±1.4)C	13.8(±1.0)D	32.3(±1.9)C	9.5(±0.2)Y	18.7(±0.6)X	25.8(±1.0)L	9.0(±0.7)Y
	10 *	7.3(±0.4)A	10.5(±0.7)E	26.3(±0.4)II	18.3(±0.7)X	20.0(±1.1)K	25.3(±0.2)I	8.5(±0.8)B
*C. famata (b)*	*0*	0(±0.0)	7.5(±0.8)A	22.5(±0.8)J	0.0(±0.0)	11.0(±0.4)G	20.8(±1.2)K	7.0(±0.4)A
	*3*	0.0(±0.0)	8.0(±0.4)B	26.0(±0.5)L	7.0(±0.1)A	11.0(±1.0)G	22.0(±0.5)J	7.0(±0.4)A
	10 *	0.0(±0.0)	7.5(±0.6)A	23.3(±0.4)M	7.0(±0.2)A	11.0(±0.5)G	23.5(±0.2)M	7.0(±0.2)A
*W. anomalus*	*0*	0 (±0)	8.0(±0.1)B	16.0(±0.4)N	0 (±0)	15.0 (±0.3)F	24.0(±1.1)M	10.0(±0.4)E
	*3*	0 (±0)	8.0(±0.1)B	14.3(±0.5)F	0 (±0)	14.0(±0.1)D	22.5(±0.2)J	9.3(±0.1)Y
	10 *	0 (±0)	8.0(±0.2)B	11.3(±0.4)G	0 (±0)	14.0(±0.2)D	17.5(±0.5)X	9.3(±0.4)Y
*C. neoformans*	*0*	13.0 (±0.8)D	14.5(±0.2)F	31.5(±0.4)O	12.5(±0.2)C	24.0(±0.5)Z	33.0(±0.7)P	8.0 (±0.4)B
	*3*	12.0(±0.4)C	13.8(±0.4)D	32.3(±1.1)P	14.0(±0.2)F	20.3(±0.6)K	29.8(±1.3)Q	9.8(±0.3)E
	10 *	13.5(±0.2)D	15.0(±1.0)F	29.8(±0.4)Q	13.0(±0.2)D	20.0(±0.2)K	30.0(±1.4)Q	9.0(±0.2)Y
*M. pachydermatis (a)*	*0*	0 (±0)	9.8(±0.3)E	39.5(±1.5)R	7.5(±0.4)A	14.0(±0.1)F	24.0(±0.5)M	8.0(±0.7)B
	*3*	0(±0)	11.8(±0.4)C	42.5(±1.1)S	8.0(±1.0)B	14.0(±0.1)F	24.8(±0.3)I	8.0(±0.6)B
	10 *	0(±0)	11.3(±0.3)G	43.5(±1.8)T	8.2(±0.2)B	14.5(±0.2)F	25.0(±0.4)I	9.3(±0.3)Y
*M. pachydermatis (b)*	*0*	0.0(±0.0)	9.8(±0.4)E	43.5(±1.3)T	7.8(+/0.4)B	15.0(±0.4)F	27.1(±1.1)II	8.5(±0.4)B
	*3*	0.0(±0.0)	12.8(±0.4)H	46.3(±1.1)V	8.8(±0.3)Y	22.0(±1.1)J	30.0(±1.4)Q	8.0(±0.4)B
	10 *	0.0(±0.0)	10.5(±0.7)E	49.8(±1.5)W	12.8(±0.4)H	16.0(±0.5)N	29.5(±0.8)Q	7.5(±0.6)A

*10 g/L BSA plus 10 g/L yeast extract, Alphabetical lettering A to X designates significant difference at *p* < 0.5 for sensitivity to test chemical. (a) and (b) denotes different strains of the same species for C. famata and M. pachydermatis.
